# Implications of Differential Age Distribution of Disease-Associated Meningococcal Lineages for Vaccine Development

**DOI:** 10.1128/CVI.00133-14

**Published:** 2014-06

**Authors:** Carina Brehony, Caroline L. Trotter, Mary E. Ramsay, Manosree Chandra, Keith A. Jolley, Arie van der Ende, Françoise Carion, Lene Berthelsen, Steen Hoffmann, Hjördís Harðardóttir, Julio A. Vazquez, Karen Murphy, Maija Toropainen, Manuela Caniça, Eugenia Ferreira, Mathew Diggle, Giles F. Edwards, Muhamed-Kheir Taha, Paola Stefanelli, Paula Kriz, Steve J. Gray, Andrew J. Fox, Susanne Jacobsson, Heike Claus, Ulrich Vogel, Georgina Tzanakaki, Sigrid Heuberger, Dominique A. Caugant, Matthias Frosch, Martin C. J. Maiden

**Affiliations:** a Department of Zoology, University of Oxford, Oxford, United Kingdom; b Department of Veterinary Medicine, University of Cambridge, Cambridge, United Kingdom; c Public Health England, London, United Kingdom; d The Netherlands Reference Laboratory for Bacterial Meningitis, Academic Medical Centre, Department of Medical Microbiology, Amsterdam, Netherlands; e Meningococcal Reference Laboratory, Scientific Institute of Public Health, Brussels, Belgium; f Neisseria and Streptococcus Reference Laboratory, Statens Serum Institut, Copenhagen, Denmark; g Department of Microbiology, Landspitali University Hospital, Reykjavik, Iceland; h Meningococcal Reference Laboratory, Madrid, Spain; i Irish Meningococcal and Meningitis Reference Laboratory, Dublin, Ireland; j National Institute for Health and Welfare, Helsinki, Finland; k Laboratory of Antimicrobial Resistance, Department of Infectious Diseases, National Institute of Health Dr. Ricardo Jorge, Lisbon, Portugal; l The Scottish Haemophilus, Legionella, Meningococcus, and Pneumococcus Reference Laboratory, Glasgow, United Kingdom; m National Reference Centre for Meningococci, Pasteur Institute, Paris, France; n Department of Infectious, Parasitic, and Immune-Mediated Diseases, Istituto Superiore di Sanità, Rome, Italy; o National Reference Laboratory for Meningococcal Infections, National Institute of Public Health, Prague, Czech Republic; p Meningococcal Reference Unit, Manchester Royal Infirmary, Manchester, United Kingdom; q National Reference Laboratory for Pathogenic Neisseria, Department of Laboratory Medicine, Clinical Microbiology, Örebro University Hospital, Örebro, Sweden; r Institut für Hygiene und Mikrobiologie, Würzburg, Germany; s National Meningococcal Reference Laboratory, National School of Public Health, Athens, Greece; t National Reference Centre for Meningococci, Institute for Medical Microbiology and Hygiene, Graz, Austria; u Department of Bacteriology and Immunology, Norwegian Institute of Public Health, Oslo, Norway

## Abstract

New vaccines targeting meningococci expressing serogroup B polysaccharide have been developed, with some being licensed in Europe. Coverage depends on the distribution of disease-associated genotypes, which may vary by age. It is well established that a small number of hyperinvasive lineages account for most disease, and these lineages are associated with particular antigens, including vaccine candidates. A collection of 4,048 representative meningococcal disease isolates from 18 European countries, collected over a 3-year period, were characterized by multilocus sequence typing (MLST). Age data were available for 3,147 isolates. The proportions of hyperinvasive lineages, identified as particular clonal complexes (ccs) by MLST, differed among age groups. Subjects <1 year of age experienced lower risk of sequence type 11 (ST-11) cc, ST-32 cc, and ST-269 cc disease and higher risk of disease due to unassigned STs, 1- to 4-year-olds experienced lower risk of ST-11 cc and ST-32 cc disease, 5- to 14-year-olds were less likely to experience ST-11 cc and ST-269 cc disease, and ≥25-year-olds were more likely to experience disease due to less common ccs and unassigned STs. Younger and older subjects were vulnerable to a more diverse set of genotypes, indicating the more clonal nature of genotypes affecting adolescents and young adults. Knowledge of temporal and spatial diversity and the dynamics of meningococcal populations is essential for disease control by vaccines, as coverage is lineage specific. The nonrandom age distribution of hyperinvasive lineages has consequences for the design and implementation of vaccines, as different variants, or perhaps targets, may be required for different age groups.

## INTRODUCTION

Neisseria meningitidis, the meningococcus, is a pathogen of global significance that causes sporadic cases and periodic epidemics and pandemics of meningitis and septicemia. The disease is associated with high mortality rates and severe sequelae in many patients who recover. Disease rates vary with age, with the highest rates for children and young adults (1–3). However, the meningococcus is usually carried asymptomatically in the nasopharynx of approximately 10% of the human population (4–7). Carriage rates in the population also vary with age, being lowest among infants and young children and rising to a peak among adolescents and young adults (8–10).

Twelve immunochemically distinct meningococcal polysaccharide capsules have been described (12), corresponding to meningococcal serogroups, but most disease is caused by meningococci expressing serogroups A, B, C, Y, W, and X (13). Worldwide invasive meningococcal serogroup distributions vary with region (3, 13); serogroups A, W, and X predominate in Africa, particularly in the “meningitis belt” region (14), whereas most disease in Western Europe is associated with meningococci expressing serogroup B and C capsules. Serogroups B and C also predominate in North and South America and in high-income countries such as New Zealand and Australia (3, 13, 15, 16). Serogroup Y disease has emerged recently as a public health concern in the United States and Canada (17, 18) and more recently in Europe (3, 11, 19, 20). The emergence of serogroup Y-associated lineages in disease and carriage populations underlines the dynamic nature of meningococcal epidemiology and population biology. These changes have implications for vaccine development and implementation.

There has been a decline in meningococcal disease incidence in Europe since 1999, decreasing from 1.9 cases per 100,000 individuals in 1999 to 0.73 cases per 100,000 individuals in 2010 (21). This decline is due in part to implementation of the meningococcal C conjugate (MCC) vaccine in a number of European countries. However, this cannot account for reductions in serogroup B disease incidence, which may be attributable to natural fluctuations. With new protein-based substitute serogroup B vaccines such as Bexsero (22) being licensed in Europe, it is important to monitor changes in the meningococcal population, particularly as herd immunity effects for this type of noncapsular vaccine are largely unknown. Outer membrane protein-based vaccines have been implemented previously in Europe, but that was in response to single-clone outbreaks (23, 24). The diversity of genotypes in a setting of endemicity represents a challenge for vaccine development and implementation. Detailed characterization of disease and carriage isolates, including the age distribution of disease-associated lineages, is essential for the improvement of disease prevention and control strategies.

## MATERIALS AND METHODS

### European meningococcal disease isolates.

Bacterial samples were obtained from European reference laboratories over 3 years, i.e., 2000 to 2002. A structured sampling program was undertaken to ensure a representative sample; laboratories that received ≤80 disease isolates per year submitted all isolates, and laboratories that processed >80 disease isolates per year sent every third isolate, with the exception of the England and Wales Meningococcal Reference Unit, which sent every tenth isolate, as the unit received >1,000 samples per year. A total of 4,183 samples were received and processed by the European Meningococcal MLST Centre (EMMC) (25), and multilocus sequence typing (MLST) was completed for 4,048 of the samples.

### MLST and sequence assembly.

MLST was performed as described previously (26, 27). Separation of the labeled extension products was carried out on a 3700 or 3730 capillary DNA analyzer (Applied Biosystems). Assembly and editing of MLST sequence data generated were carried out using STARS (http://sourceforge.net/projects/stars) and Staden software, with Pregap 4 version 1.3 and Gap version 4.7 (28). For each isolate, sequences for each of the seven loci were assigned allele numbers through interrogation of the Neisseria MLST database (http://pubmlst.org/neisseria). Allelic profiles were assigned a sequence type (ST) and a clonal complex (cc) using the database.

### Data analysis.

MLST data were combined with age information collected separately for the isolates by the European Meningococcal Epidemiology Centre (EMEC)/European Union Invasive Bacterial Infections Surveillance Network (EU-IBIS) (29) when such data were available. Multinomial regression analyses were performed using Intercooled Stata 12.0 for Windows (StataCorp, College Station, TX). Simpson's index of diversity (*D*) was used to determine ST diversity by age group. Calculation of discriminatory indices was performed as described previously (30). The value of the index ranges from 0 to 1, with values nearer to 1 indicating greater diversity. The 95% confidence intervals (CIs) for these indices were calculated as described by Grundmann et al. (31). The evenness value (*E*) is a measure of the relative abundance of the different genotypes making up the richness, i.e., the number of genotypes in a population sample (such as a country). The value ranges from 0 to 1, with values nearer to 1 indicating more even contributions of the genotypes to the overall sample. According to the method described by Robinson et al. (32), the value is calculated as the ratio of the effective number of clones (*Se*), in this case STs, to the total number of clones (genotypes) (*S*), i.e., the richness of the sample. *Se* and *E* increase as the numbers of isolates of each clone become more equal.

## RESULTS

**FIG 1:**
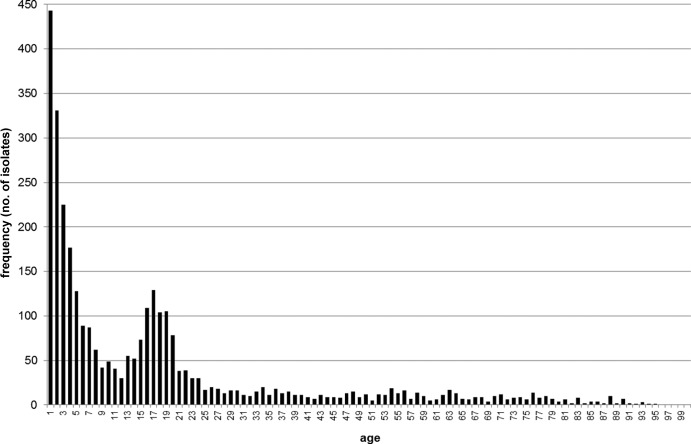
Age distribution of disease isolates in the European meningococcal disease collection.

**FIG 2:**
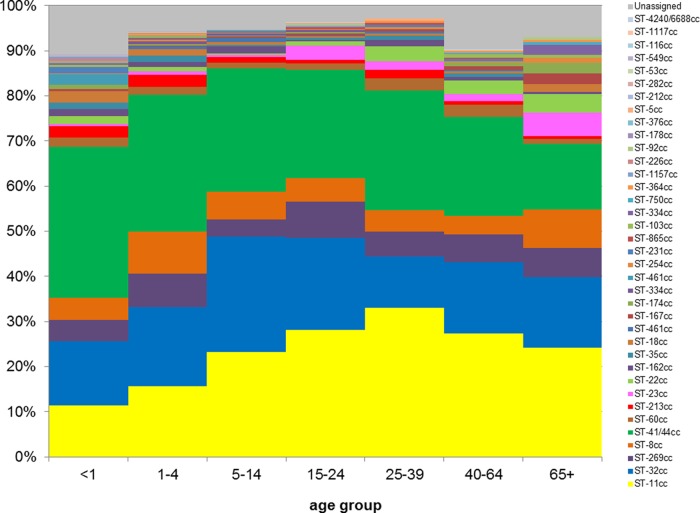
Age distribution of common clonal complexes according to their proportions in the European meningococcal disease collection.

**FIG 3:**
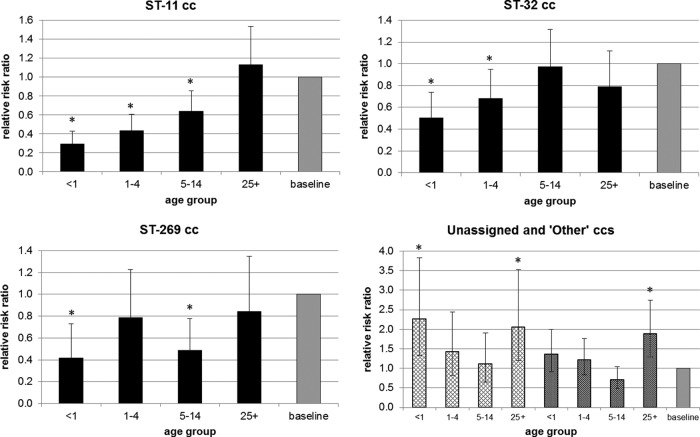
Relative risk ratios (RRRs) of disease by age group for ST-11, ST-32, and ST-269 ccs, unassigned STs (hatched bars), and other ccs (dark gray bars). ‘Other' ccs include ST-213, ST-23, ST-22, ST-60, ST-35, ST-461, ST-162, ST-18, ST-174, ST-334, ST-167, ST-364, ST-254, ST-103, ST-865, ST-231, ST-750, ST-1157, ST-53, ST-5, ST-226, ST-198, ST-212, ST-92, ST-1136, ST-178, ST-282, ST-37, ST-376, ST-1117, ST-116, ST-175, ST-4240/6688, and ST-549 ccs. *, significant RRR, compared with the baselines of 15 to 24 years and the ST-41/44 cc.

Patient age data were available for 3,226 (87.1%) of the 3,705 EU-IBIS epidemiological data records that were able to be harmonized with the EUMenNet data, and complete MLST data were available for 3,147 of those records (Fig. 1). An overall χ^2^ test demonstrated a cc-age effect (*P* < 0.001) (Table 1 and Fig. 2 and 3). The age group of 15 to 24 years and the ST-41/44 cc were used as baselines for multinomial regression analyses. The following significant age effects were observed: (i) subjects <1 year of age experienced lower risk of ST-11 cc disease (relative risk ratio [RRR], 0.29 [95% CI, 0.20 to 0.43]), ST-32 cc disease (RRR, 0.50 [95% CI, 0.35 to 0.74]), and ST-269 cc disease (RRR, 0.42 [95% CI, 0.24 to 0.73]) and higher risk of disease due to unassigned STs (RRR, 2.25 [95% CI, 1.33 to 3.83]); (ii) subjects 1 to 4 years of age experienced lower risk of ST-11 cc disease (RRR, 0.44 [95% CI, 0.31 to 0.60]) and ST-32 cc disease (RRR, 0.68 [95% CI, 0.49 to 0.95]); (iii) subjects 5 to 14 years of age were less likely to experience ST-11 cc disease (RRR, 0.64 [95% CI, 0.48 to 0.86]) and ST-269 cc disease (RRR, 0.49 [95% CI, 0.31 to 0.78]); and (iv) subjects ≥25 years of age were more likely to experience disease due to other ccs (i.e., less common ccs that were grouped together for the purposes of this analysis (see Table 1 and Fig. 2 for details) (RRR, 1.88 [95% CI, 1.29 to 2.74]) and unassigned STs (RRR, 2.05 [95% CI, 1.20 to 3.53]). A number of countries implemented the MCC vaccine during the time period of the study (United Kingdom in 1999, the Republic of Ireland in 2000, and the Netherlands in 2002). To account for the possible effects of the implementation on ST-11 cc disease risk, the regression analysis was repeated without isolates from these countries (and those from 2002 from the Netherlands). The lower risk of ST-11 cc disease remained for subjects <1 year of age (RRR, 0.36 [95% CI, 0.24 to 0.57]) and subjects 1 to 4 years of age (RRR, 0.49 [95% CI, 0.35 to 0.69]), with weaker evidence for subjects 5 to 14 years of age (RRR, 0.74 [95% CI, 0.52 to 1.05]).

**TABLE 1:** Age distribution among the most common clonal complexes in the European meningococcal disease collection

Clonal complex	No. (%) of isolates per age group					Missing data (no. [%] of isolates)	Total no. of isolates
	<1 yr	1–4 yr	5–14 yr	15–24 yr	≥25 yr		
ST-41/44	143 (14)	253 (25)	155 (15)	161 (16)	138 (14)	164 (16)	1,014
ST-11	49 (5)	131 (15)	132 (15)	189 (21)	183 (20)	219 (24)	903
ST-32	61 (9)	146 (21)	145 (21)	136 (19)	92 (13)	127 (18)	707
ST-8	21 (8)	78 (29)	35 (13)	35 (13)	36 (13)	68 (25)	273
ST-269	20 (8)	63 (25)	22 (9)	54 (21)	39 (15)	58 (23)	256
Others^*a*^	88 (14)	117 (19)	48 (8)	71 (11)	114 (18)	193 (31)	631
Unassigned	46 (17)	48 (18)	31 (12)	24 (9)	43 (16)	72 (27)	264
Total	428	836	568	670	645	901	4,048

a ‘Other' ccs include ST-213, ST-23, ST-22, ST-60, ST-35, ST-461, ST-162, ST-18, ST-174, ST-334, ST-167, ST-364, ST-254, ST-103, ST-865, ST-231, ST-750, ST-1157, ST-53, ST-5, ST-226, ST-198, ST-212, ST-92, ST-1136, ST-178, ST-282, ST-37, ST-376, ST-1117, ST-116, ST-175, ST-4240/6688, and ST-549 ccs.

There was a range of diversity of STs accounting for disease according to age group, with the lowest diversities found in the 25- to 44-year (*D* = 0.899) and 15- to 24-year (*D* = 0.914) age groups. The highest diversity values were found in the <1-year (*D* = 0.973) and 1- to 4-year (*D* = 0.959) age groups. There was significantly greater diversity among subjects <5 years of age versus all other age groups except the ≥45-year age group. There was also varying evenness of STs among the different age groups. The lowest evenness value was found in the 15- to 24-year age group (*E* = 0.081), and the highest value was found in the <1-year age group (*E* = 0.180). This indicates that, in addition to there being lower diversity among 15- to 24-year-olds, the distribution of genotypes was much less even, demonstrating that disease in that age group was dominated by particular STs and ccs. Other age groups, such as the <1-year age group, had more even distributions of STs associated with disease.

## DISCUSSION

Several protein-based vaccines to prevent serogroup B meningococcal disease are in various stages of development (33), with one, Bexsero, recently being licensed in Europe. Molecular epidemiology has become an essential part of this development and implementation. Much analysis of the distribution of the four antigens present in this vaccine has been carried out in recent years, in collections of disease-associated serogroup B-expressing organisms (34–36). Based on the meningococcal antigen typing system (MATS) assay, there is predicted coverage of 78% of disease-associated serogroup B-expressing meningococci in Europe (36). As Bexsero antigen data are not available for this collection, an estimate based on previous studies (34) showing strong associations of antigen and cc would give a baseline coverage of almost 50% for this data set regardless of serogroup (with ST-41/44 cc, ST-32 cc, and ST-8 cc serving as proxies for NHBA-2 and P1.4, fHbp-1.1, and NadA-3.8, respectively). Representative, well-sampled, isolate collections such as the EUMenNet collection provide insights into the dynamics of the population biology of the meningococcus and facilitate the planning and implementation of interventions, including immunization programs. Since serogroups and other vaccine antigens are known to be associated with ccs, the differences we observed in the distribution of ccs with age concur with, and extend, previous work in both Europe and North and South America. Studies have indicated a higher prevalence of non-BC serogroups in older age groups, with serogroup B disease being proportionally the greatest among subjects <1 year of age (15, 16, 19, 21, 37–39). The present analysis also indicated that the diversity of disease-causing meningococci was higher in the youngest and oldest age groups and that ccs other than the major hyperinvasive lineages and unassigned STs were more associated with older age groups.

The relatively lower prevalence of ST-11 cc and ST-269 cc among individuals <14 years of age was consistent with findings in Canada, where there was an association of ST-269 cc in 11- to 40-year-olds and many fewer cases among <1-year-olds (40). Also, a study of meningococcal disease in Poland over 10 years demonstrated a significantly higher frequency of ST-11 cc-associated disease among individuals >5 years of age (41). As with ST-11 cc and ST-269 cc, the serogroup B-associated ST-32 cc was less likely to affect subjects <4 years old. These findings are consistent with the presence of the meningococcal disease-associated (MDA) phage, a candidate virulence factor associated with adolescent disease, in these lineages (42).

The nonrandom variation in age distributions of meningococcal lineages has several consequences. It demonstrates that the different ccs have different phenotypes in terms of disease association and probably carriage. Genotypes that are thought to be comparatively less invasive, along with those not assigned to a cc, were more likely to affect the very young or relatively old (<1 or >65 years of age), which may be due to these cohorts being vulnerable to higher rates of disease caused by less-invasive meningococci, perhaps as a consequence of poorer immune responses. Niche competition with commensal organisms such as Neisseria lactamica, which has its highest rates of carriage in 1- to 2-year-olds, may have an influence on meningococcal carriage and thus potentially disease (43, 44). Immunologically mature individuals such as older adolescents and young adults may more easily clear less-virulent strains but then be more susceptible to more-virulent strains such as ST-11 cc, which may have shorter durations of carriage. Some of these differences may be due to behavioral factors or potential virulence factors such as the MDA phage, which may affect the expression of certain genes. These differences in age associations may have consequences for the design and implementation of vaccines, as different variants or perhaps targets may be required for different age groups. Like other well-studied vaccine candidate antigens, such as PorA and FetA, those included in newly developed vaccines such as Bexsero have associations with clonal complexes (34, 35). Therefore, it is expected that they will also have different age distributions, which may have consequences for vaccine implementation.

Given the diversity of the meningococcal population, a relatively small number of genotypes are associated with disease. In Europe, five ccs (ST-41/44, ST-11, ST-32, ST-8, and ST-269 ccs) accounted for 77% of the disease isolates. These hyperinvasive lineages are a subset of those observed globally and represent a minority of carried meningococci (45–47). The prevalence of particular hyperinvasive lineages in carriage changes over time, and this is reflected in the rates of disease that they cause. For instance, in the past decade previously rare serogroup Y-associated lineages increased in prevalence, in both disease and carriage, in Europe (3, 11, 19, 20, 48–51). It is therefore necessary to maintain surveillance to identify changes in the distribution of types, including the emergence of new clones and possible capsule-switching events in the face of immunization campaigns. This should include monitoring changes in genotype distributions, in disease and carriage, according to age group. Initiatives such as the Meningitis Research Foundation Meningococcus Genome Library are valuable resources that will allow such analyses using the latest molecular epidemiological tools. In the absence of serogroup B conjugate polysaccharide vaccines, the control of meningococcal disease will rely on the implementation of protein-based vaccines, the coverage of which will vary as changes occur in the circulating meningococcal populations over time. Any implementation of new vaccines, such as Bexsero, that target proteins that are derived from particular serogroup B meningococci but may be shared by strains belonging to other serogroups, will therefore require intensive epidemiological surveillance.
